# Epidemiological Study of Thogoto and Dhori Virus Infection in People Bitten by Ticks, and in Sheep, in an Area of Northern Spain

**DOI:** 10.3390/ijerph17072254

**Published:** 2020-03-27

**Authors:** Lourdes Lledó, Consuelo Giménez-Pardo, María Isabel Gegúndez

**Affiliations:** Departamento de Biomedicina y Biotecnología, Universidad de Alcalá, 28801 Alcalá de Henares, Spain; consuelo.gimenez@uah.es (C.G.-P.); isabel.gegundez@uah.es (M.I.G.)

**Keywords:** epidemiology, tick-borne viruses (TBVs), Thogoto virus, zoonosis

## Abstract

There is little information on Thogoto virus (THOV) and Dhori virus (DHOV)infection in Spain. A total of 283 serum samples from 150 human subjects (78 males, 72 females) bitten by ticks, as well as samples from 120 sheep (one per animal), were studied by immunofluorescence assay. All human and animal subjects were from the province of Palencia in northern Spain. Eight human subjects had antibodies against THOV (seroprevalence: 5.3%) and six had antibodies against DHOV (seroprevalence: 4%); titers ranged between 1/32–1/256 and 1/32–1/128, respectively. No significant differences were seen in seroprevalence in terms of gender or age, although people with antibodies were significantly more likely to have had contact with livestock for professional reasons. One subject with an acute infection had IgM antibodies to both viruses and seroconverted to IgG. For the sheep, 24 serum samples were positive for antibodies to THOV (seroprevalence: 20%) and 32 for antibodies to DHOV (seroprevalence: 26.8%); titers ranged between 1/16 and 1/128. The seroprevalence of both viruses was significantly higher in animals < 4 years of age. Together, these results reveal the circulation of DHOV and THOV in humans and sheep in the province of Palencia. Sheep might be used as indicators of the presence of these organisms.

## 1. Introduction

Ticks can transmit viruses responsible for severe emerging and re-emerging infectious diseases —some with a significant impact on public health. Thogoto virus (THOV) and Dhori virus (DHOV) (Family: Orthomyxoviridae; Genus: *Thogotovirus*) are both transmitted by ticks to humans, and occasionally cause problems ranging from benign febrile symptoms to meningoencephalitis [1]. THOV was recorded as the possible agent responsible for the death of a man in Kansas who had a history of tick bites. After suffering fever and fatigue for 11 days, he died due to multiorgan failure [2]. DHOV has been detected in laboratory workers in Russia who were accidentally infected via an aerosol during culture preparation; they developed a fever that lasted for 2–4 days, which was probably associated with an encephalitic reaction [3]. Some authors suggest that both viruses are transmitted from person to person [4], but so far there has been no evidence to substantiate this.

Animal reservoirs of THOV and DHOV appear to include banded mongooses (*Mungos mungo*) [5], rodents [6], dromedaries, and domesticated animals such as cattle and other livestock [7]. However, the natural transmission cycle probably involves many other species [4,8,9]. For example, THOV has been serologically detected in domesticated mammals, rats, and humans in Egypt [10], and Moore et al. isolated THOV from people in Nigeria [1].

Tick-borne viruses affecting sheep and goats can cause locally important diseases associated with significant production losses. Certainly, THOV can be transmitted by *Ixodid* ticks and has been associated with miscarriage in sheep [9,11]. A strain of THOV has also been isolated from *Ixodid* ticks (*Rhipicephalus* spp.) on cattle in Kenya [12]. The same virus was also isolated from *Amblyomma variegatum*, *Boophilus annulatus*, and *Hyalomma nitidum* ticks during the République Centrafricaine’s dry season (December to February) [13]. DHOV has been isolated from *Hyalomma marginatum* ticks on livestock in southern Portugal [14], and THOV has been isolated from *Rhipicephalus sanguineus* ticks on goats from Vila Viçosa in eastern-central Portugal [15]. The latter THOV were shown to be serologically identical to viruses isolated from *R. bursa* ticks in Sicily [16]. L’vov et al. isolated DHOV from *Hyalomma marginatum* ticks on hares in Astrakhan (in the Volga delta) [17]. In Kyoto, Japan, THOV has been recently isolated from *Haemaphysalis longicornis* [18], and *Amblyomma americanum* has been shown to be a vector of Thogoto virus to humans in the USA [19]. The first report of DHOV from Kenya implicated *Amblyomma gemma* as a vector in this region [20]. Surveillance studies have since demonstrated the circulation of DHOV in parts of the eastern and northeastern provinces of Kenya in *R. pulchellus* [21].

Spain’s geographical proximity to Africa, and its different climates and ecological conditions, mean that both THOV and DHOV could be present [22]. To date, however, epidemiological and clinical studies on these viruses in Spain have been very scarce. The present work examines the seroprevalence of THOV and DHOV of people bitten by ticks, and in sheep, in an area of northern Spain.

## 2. Materials and Methods 

### 2.1. Study Area

**Figure 1:**
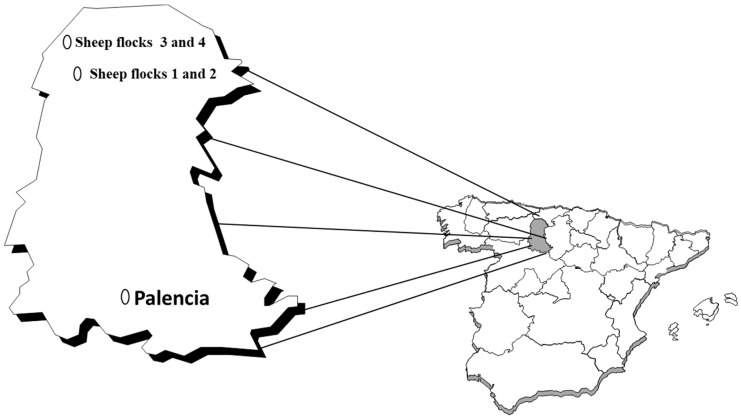
Palencia province, and the geographical distribution of the examined sheep.

This work was performed in the Spanish province of Palencia (Figure 1), a mainly rural area with numerous isolated villages, the main activities of which are forestry, agriculture, and stock-raising.

### 2.2. Serum Samples

The serum samples used in this work form part of our group’s frozen (−20 °C) serum collection. Over a period of 7 years, 283 samples were collected at primary healthcare centers from 150 patients (78 males (52%), 72 females (48%); age range 3–85 years, median age 38 years (IQR: 23–56 years)). Blood was taken once (at first presentation) from 51 patients (34%), twice from 65 patients (43.3%) (at first presentation and 30 days later), and three times from 34 patients (22.6%) (at first presentation, 30 days later, and at 90 days). All patients provided information on their age, sex, occupation, place of residence, symptomology, contact with domesticated animals (including livestock and pets), and tick bites.

Serum samples were also collected from 120 sheep (one per animal) belonging to four flocks in two localities in the north of the province of Palencia (Figure 1). Among these animals, 48 were <4 years of age, 60 were aged 4–5 years, and 12 were >5 years old (age range 2–12) years; the median age was 4 years (IQR: 3–5 years). The median age of the animals in flock 1 was 4.03 years, in flock 2 (from the same locality as flock 1) it was 3.7 years (the mean age of these flocks together was 3.88 years), in flock 3 it was 3.3 years, and in flock 4 (from the same locality as flock 3) it was 4.9 years (the mean age of these last two herds was 4.13 years). Blood was obtained (from anaesthetized sheep by jugular venipuncture) and allowed to clot.

All patients (or their legal guardians in the case of minors) gave their informed consent to be included in this study, in compliance with the ethical standards of the Ethics Committee of the University of Alcalá and the Declaration of Helsinki 1975 (as revised in 2013). Permission to take and study animal samples was obtained from the Regional Government of Castilla y León in compliance with current legislation (Protocol number 06.01.017.006). The study protocol was approved by the Ethics Committee of the University of Alcalá (Protocol number CEI 2011034).

### 2.3. Immunofluorescence

Sera were examined by indirect immunofluorescence assay (IFA), as described by Filipe et al. [23]. THOV (strain POTi503 (SM 10) ) and DHOV (DHOV POTiP12 (SM1) ) were used as antigens. These were propagated in Vero E6 cells (ATCC CRL 1586) and fixed on spot slides. For human samples, the fluorescein-labeled conjugate used was rabbit anti-human IgG and IgM (Sigma, St Louis, MO, USA); for sheep samples, it was FITC donkey anti-sheep IgG (Sigma). Positive and negative control sera were included (gifts from the Centro de Estudos de Vectores e Doenςas Infecciosas, Portugal). Titers of ≥1/32 (IgG) and 1/16 (IgM) were considered positive for humans; 1/16 was considered positive for sheep.

### 2.4. Statistical Analysis

Differences in proportions were analyzed using the χ^2^ or Fisher exact test as required. Significance was set at *p* < 0.05.

## 3. Results

Eight of the human subjects (seroprevalence: 5.3%) showed IgG antibodies against THOV. Six were male (seroprevalence: 7.7%) and two were female (seroprevalence: 2.7%) (*p* = 0.278 Fisher exact test). Six patients (seroprevalence: 4%) had antibodies to DHOV. Four were male (seroprevalence: 5.1%) and two females (seroprevalence: 2.7%) (*p* = 0.682 Fisher exact test). Titers ranged between 1/32 and 1/256 for antibodies to THOV, and 1/32 and 1/128 for antibodies to DHOV. Six patients had IgG antibodies to THOV and DHOV (Table 1).

Taking the results for both viruses together, the seropositive patients were aged 5–71 years (Table 2) (median: 39 years; IQR: 26.5–59.75). No significant differences in seroprevalence were seen with respect to age group (χ^2^ = 2.372; *p* = 0.498).

Most patients had been bitten in the spring (71 people (47.3%) ) or autumn (29 people (19.3%) ). Table 3 shows the seroprevalence of infection in terms of patient occupation. All seropositive patients had contact with dogs and cats; 22% of the studied professionals who had contact with cattle, sheep, and goats (i.e., farmers, stock-raisers, shepherds, and veterinarians) were seropositive (χ^2^ = 3.860; *p* = 0.049 compared to those with no professional contact with livestock). Retirees (positive and non-positive) had no contact with livestock in their current life or in their pre-retirement professional life.

One patient who provided two samples (a construction worker who consulted for several tick bites on his head) showed evidence of an asymptomatic acute infection. At one point, he was IgM-positive (IgM seroprevalence 0.6%) and IgG-positive for both viruses (Table 1); the IgG titer rose between the first and second serum sample, while the IgM titer decreased to negative by the second sample. 

Twenty-four of the sheep serum samples had antibodies to THOV (seroprevalence: 20%), and 32 had antibodies to DHOV (seroprevalence: 26.6%) (χ^2^ = 1.490; p = 0.222). Eleven animals had antibodies against both viruses (seroprevalence: 9.2%). The titers of the seropositive samples ranged between 1/16 and 1/128. Seropositive animals were detected in all four herds, but in flocks 1 and 2 the seroprevalence of THOV and DHOV was higher than in flocks 3 and 4 (χ^2^ = 5.208, p = 0.022; χ^2^ = 8.352, p = 0.003, respectively), despite them all being geographically quite close to one another. Table 4 shows the distribution of seroprevalence by flock, while Table 5 does so by age.

Animals under 4 years of age returned significantly higher seroprevalence values for both viruses (THOV: χ^2^ = 4.074, *p* = 0.043; DHOV: χ^2^ = 9.204, *p* = 0.010, compared to older animals).

## 4. Discussion

Ticks can harbor and transmit viruses from wild animals to humans and livestock. The ticks themselves become infected by feeding on viremic hosts, and by co-feeding with infected ticks [19,24,25]. *Ixodid* ticks generally feed once at each developmental stage (larva, nymph, and adult) and thus have only one opportunity per stage to transmit viruses (inter-stadial transmission). Recently, Talactac et al. [18] showed that the co-feeding of infected adults and naive nymphs can lead to the latter becoming adults able of transmitting THOV to mice.

Little attention has been paid to THOV and DHOV, since they only occasionally cause disease in humans, and usually flu-like symptoms are the only problem. However, in severe cases, the central nervous system may be affected. In Europe, no clinical cases have been reported, even though both viruses have been isolated from people [14,15], and in the USA, only one likely—and fatal—clinical case has ever been recorded [2]. A non-fatal case of meningoencephalitis and hepatitis caused by THOV was reported from Nigeria in 1975 [1].

The present results reveal the presence of THOV and DHOV antibodies in humans and sheep from the study area. This is the first time THOV and DHOV seroprevalence figures have been reported for individuals bitten by ticks in Spain. These results in humans are interesting, since they cover an extended period of time and include people exposed to these viruses via their work. For both viruses, the present seroprevalence values were higher than those reported for places in Portugal (1% for THOV and 0.8% for DHOV [23]), while the seroprevalence for DHOV was less than that reported from Astrakhan (4–9%) [26].

No differences in seroprevalence were seen with respect to sex or age, similar to that reported in the above studies. The highest percentages of human seropositive results were observed among farmers and/or stock-breeders. This agrees with the findings reported by Filipe et al. [23] and can be attributed to these persons’ more frequent exposure; their professions mean they have close contact with domestic and peridomestic animals.

The detection of a patient with serological evidence of acute infection is interesting. The patient had no symptoms, but this is commonly the case.

The present results also confirm THOV and DHOV infection in sheep, with seroprevalence higher in younger animals. This might be explained in that, in this region, older animals are taken out to pasture less often.

The difference in prevalence detected between the different herds of the two localities (geographically close) is very interesting. The reasons can be varied, from differences in the hygienic conditions of the enclosures where the animals live, differences in the stable, and/or coexistence with other animals. Also, since ticks infest migratory birds, they could possibly disseminate THOV and DHOV over a wide geographic range [27]. The transmission of these viruses to different mammalian species, the inter- and intrastadial transmission of these viruses within the vectors, the lack of the need for viral replication before re-transmission in the saliva of co-feeding ticks [28,29], and possible differences in infection rates depending on ecological conditions, suggest something of the potential of THOV and DHOV to spread. Sheep may provide a useful sentinel species for their surveillance.

When patients present with tick bites, medical professionals should consider possible infection with THOV and DHOV, especially among those who work with livestock. In addition, further work on the epidemiology of these viruses is warranted to increase information of other risk factors at home related to the diseases and to know the vectors (ticks involved) and reservoir animals in our environment.

## 5. Conclusions

This study is the first to detect antibodies against the studied infectious agents in people bitten by ticks, as well as in livestock (sheep), from Spain. Sheep may provide a useful sentinel species for their surveillance. When patients present with tick bites, medical professionals should consider possible infection with THOV and DHOV, especially among those who work with livestock.

## Figures and Tables

**Table 1:** Human serum titers for the studied microorganisms. THOV: Thogoto virus; DHOV: Dhori virus.

Subjects	IgG		IgM	
	THOV	DOHV	THOV	DHOV
1	1/32	1/32	-	-
2	1/32	-	-	-
3	1/32	1/32	-	-
4	1/64	1/64	-	-
5 *	1/256	1/128	1/16	1/16
6	1/32	1/32	-	-
7	1/32	-	-	-
8	1/32	1/32	-	-

* This subject showed seroconversion to IgG.

**Table 2:** Seroprevalence with respect to age.

Age Groups.	Nº (%)
0–10 years	1 (6.6)
11–20 years	0 (0)
21–30 years	1 (5.5)
31–40 years	3 (11.1)
41–50 years	0 (0)
51–60 years	1 (5.8)
61–70 years	1 (7.1)
71–80 years	1 (10)
81 or more years	0 (0)

**Table 3:** Seroprevalence with respect to subject occupation.

Subject Occupation	THOVN º (%)	DHOVN º (%)
Farmer	1 (5.8)	1 (5.8)
Livestock	1 (12.5)	1 (12.5)
Pastor	2 (28.6)	1 (14.28)
Forestry worker	1 (25)	0 (0)
Construction worker	1 (12.5)	1 (12.5)
Retiree	1 (4.76)	1 (4.76)
Unspecified profession (mostly children under six years of age)	1 (16.66)	1 (16,66)
Student	0 (0)	0 (0)
Administrative worker (clerks, lawyers, businesspeople)	0 (0)	0 (0)
Service sector (shopkeepers, waiters, cooks, mechanics, maintenance)	0 (0)	0 (0)
Veterinarian	0 (0)	0 (0)
Health (doctors and nurses)	0 (0)	0 (0)
Housewife	0 (0)	0 (0)

**Table 4:** Seroprevalence and antibody titers for the different flocks.

Sheep Flock	Nº of sera	Pos. (%)	Titers			
**Sheep flock (THOV)**			**1/16**	**1/32**	**1/64**	**1/128**
1	30	8 (26.6%)	1	-	5	2
2	30	9 (30%)	3	1	1	4
3	30	5 (16.6)	3	1	1	-
4	30	2 (6.9%)	1	1	-	-
**Sheep flock (DHOV)**			**1/16**	**1/32**	**1/64**	**1/128**
1	30	7 (23.3%)	3	1	3	-
2	30	16 (53.3%)	6	5	5	-
3	30	7 (23.3%)	2	4	1	-
4	60	2 (6.9%)	2	-	-	-

**Table 5:** Seroprevalence with respect to animal age.

Age Groups	THOV *N* (%)	DHOV *N* (%)
<4 years	15 (31.2)	20 (41.6)
4–5 years	9 (15)	10 (16.6)
>5 years	0 (0)	2 (16.6)
